# The Utilization of Three-Dimensional Printing in Creating a Surgical Instrument: An Areola Cookie Cutter

**DOI:** 10.1093/asjof/ojac055

**Published:** 2022-06-24

**Authors:** Waleed Burhamah, Solaiman M Alshawaf, Sabika Alwazzan, Sarah AlYouha, Salman Al-Sabah

**Affiliations:** Division of Plastic & Reconstructive Surgery, Jaber Al Ahmad Al Sabah Hospital, Kuwait City, Kuwait; Division of Plastic & Reconstructive Surgery, Jaber Al Ahmad Al Sabah Hospital, Kuwait City, Kuwait; Department of General Surgery, Jaber Al Ahmad Al Sabah Hospital, Kuwait City, Kuwait; Department of Surgery, Faculty of Medicine, Kuwait University, Kuwait City, Kuwait; Department of Surgery, Faculty of Medicine, Kuwait University, Kuwait City, Kuwait

## Abstract

Three-dimensional (3D) printing is a rapidly evolving technology with many applications in the medical field. It involves printing solid objects from a digital file. In this paper, we describe our experience with the use of 3D printing in creating an areola cookie cutter that is compatible with sterilization. The objective of this study is to explore accurate and cost-effective methods of producing patient-specific areola cookie cutters. Auto computer-aided design (CAD) 3D software was used to design a digital model that was subsequently converted to a standard tessellation language (STL) file. The models were printed with the Formlabs Form 3+ SLA printer (Somerville, MA) using a resin material. Washing and curing were then performed followed by autoclave sterilization of the models. A total of 3 areola cookie cutters were created, each with different sizes (33, 38, and 42 mm) using resin material (Formlabs BioMed Clear Resin; Somerville, MA). All 3 models were able to withstand autoclave sterilization. The use of 3D printing has proven to be a valuable tool in Plastic surgery. We describe our experience of designing and producing an areola cookie cutter using a 3D printer; our model is compatible with the process of sterilization. We emphasize the advantages of a quick production time and accuracy in design.

Three-dimensional (3D) printing, or rapid prototyping, is an additive manufacturing process that allows for the creation of solid objects from a digital file, and it is a relatively new technology that has found many applications in various fields.^1^ The utilization of 3D printing in the medical field has been receiving a growing recognition; it has played a pivotal role in advancing various aspects of the field since its inception. One of its well-recognized uses is in the preoperative planning of complex cases, whereby radiological images, eg, from a computerized tomography (CT) scan, are converted into standard tessellation language (STL) in a process called “segmentation,” and this is then translated by the 3D printer into a model that can be used for planning the surgery.^1-3^ This has been shown to reduce operation time.^4^ Additionally, 3D printing has also been used to create prosthetics and implants, eg, orbital prosthesis, which are accustomed to patients’ specifications.^1,5,6^ One of its overlooked uses is in the creation of surgical instruments. Successful attempts have been made to design, print, sterilize, and use surgical instruments.^2,7-10^ In this paper, we describe our experience in designing and 3D printing an areola cookie cutter.

## METHODS

**Figure 1.**
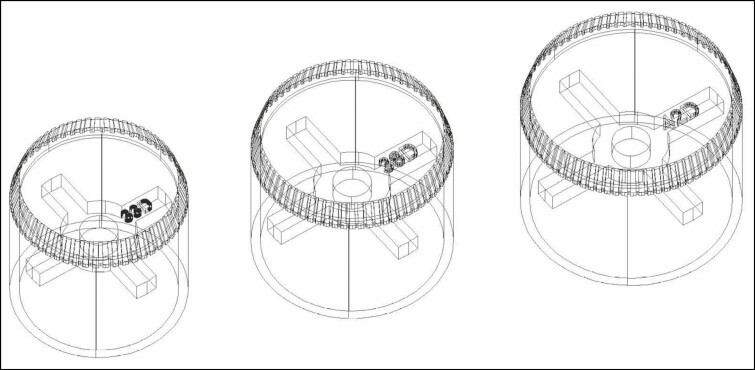
A digital model of the cookie cutter in 3 sizes (33, 38, and 42 mm) created using auto computer-aided design three-dimensional (CAD 3D) software.

**Figure 2.**
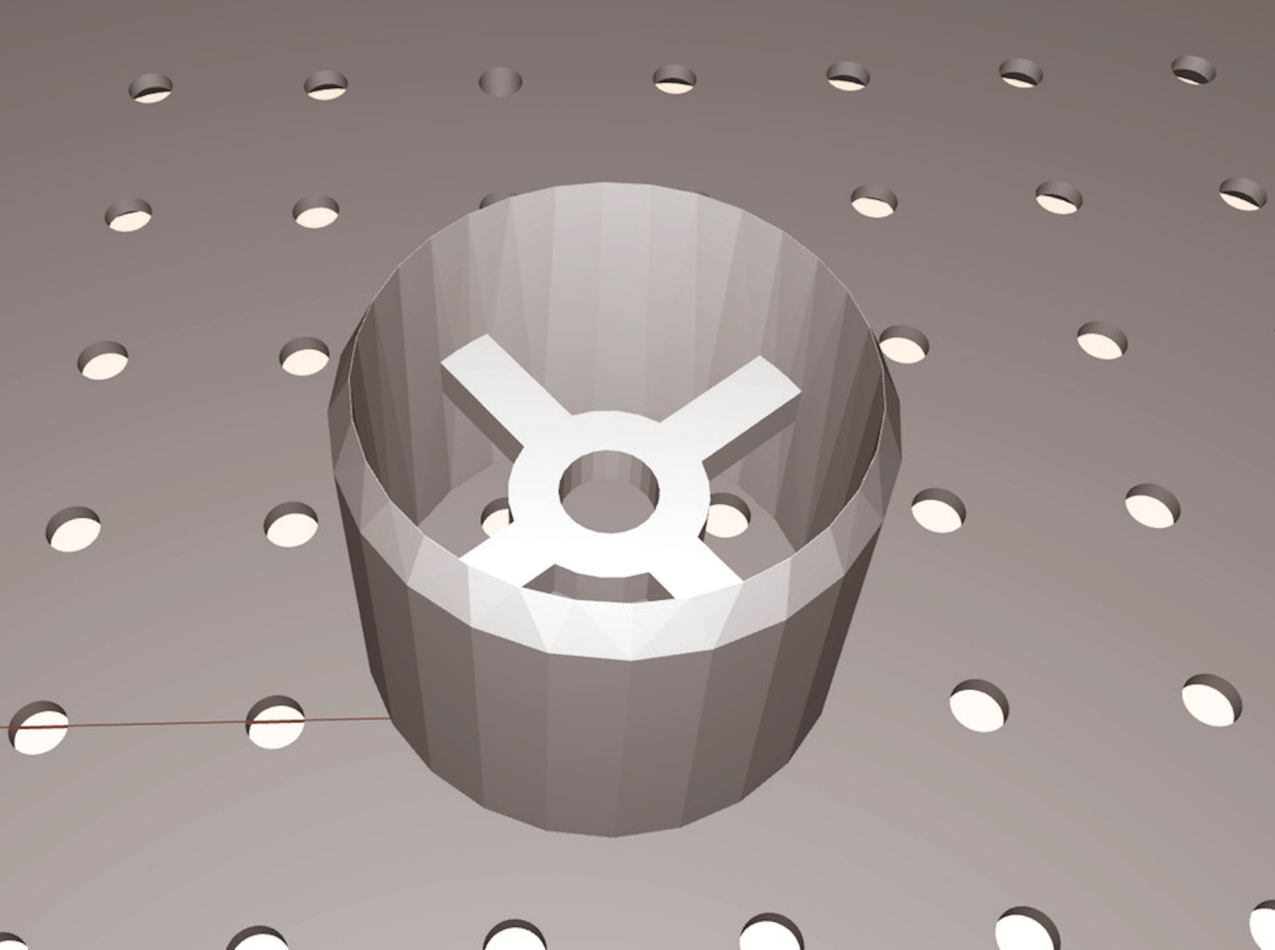
The STL file format of the digital models of the cookie cutters. STL, standard tessellation language.

Auto computer-aided design (CAD) 3D software was used to design and develop a digital model of the areola cookie cutter (Figure 1). Three different sizes (33, 38, and 42 mm) were created. The digital models were then converted to an STL file format (Figure 2) and printed using Formlabs Form 3+ SLA Printer (cost $3750US) (Figure 2). The models were printed using BioMed Clear Resin (Formlabs, Somerville, MA).^11^ The designing phase lasted 2 to 3 hours, while the total printing time lasted 4 hours. Only 45 mL of resin was used in the printing process of the 3 models (1 L of resin costs $349US).

The models were then washed using 70% isopropyl alcohol for 5 minutes using the Formlabs Form Wash device (Somerville, MA). Curing was then performed using the Formlabs Form Cure device (Somerville, MA) for 60 minutes under 60 degrees, in order to reach the greatest potential strength and optimum color. Sterilization of the models was then performed using an autoclave under 121°C for 30 minutes. To ensure successful sterilization, the printed models were directly applied to a blood agar plate and a chocolate agar plate. The agar plates were then incubated at 35°C ± 2°C in oxygen and carbon dioxide environments, respectively. No bacterial growth was detected after 48 h. Additionally, Sabouraud agar was used to detect fungal growth. After 7 days of incubation, no fungal growth was detected.

## RESULTS

**Figure 3.**
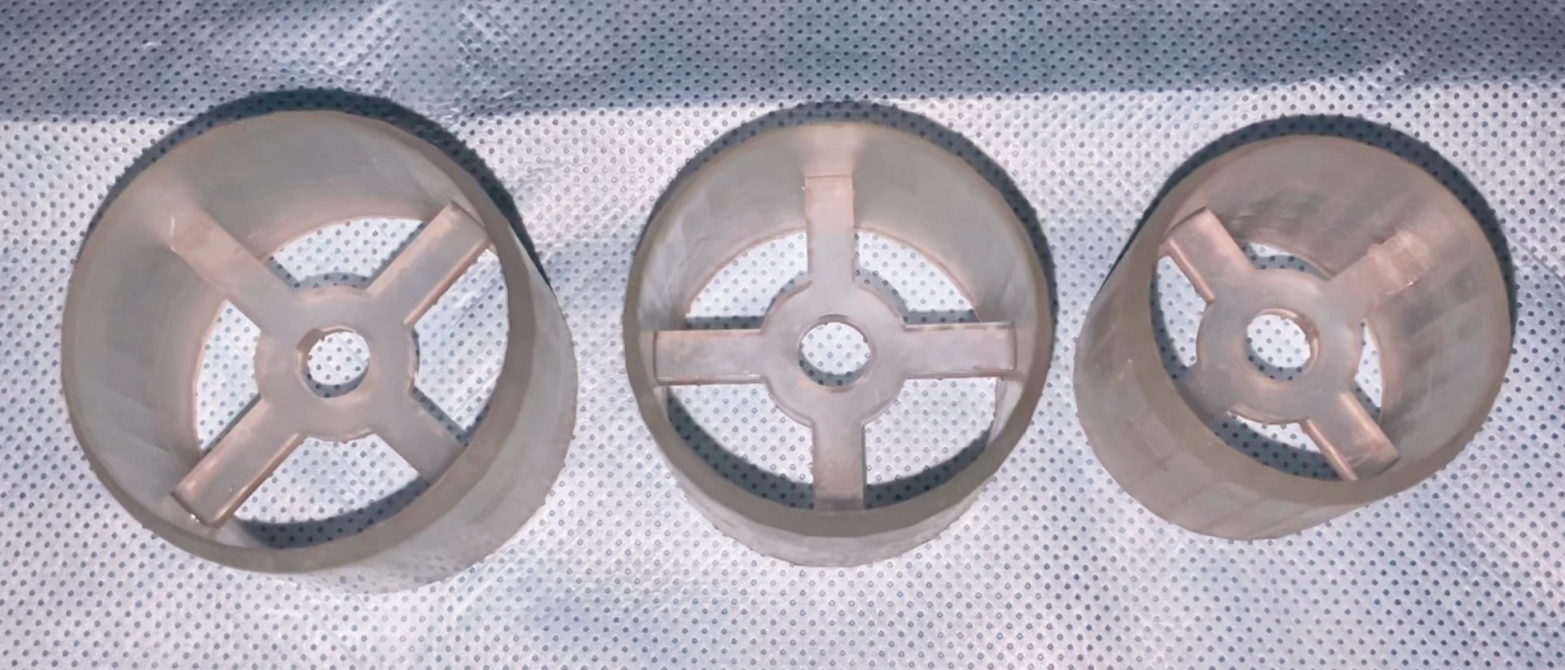
The three-dimensional printed models of cookie cutters using BioMed Clear Resin material (Formslab, Somerville, MA) in 3 sizes (33, 38, and 42 mm).

**Figure 4.**
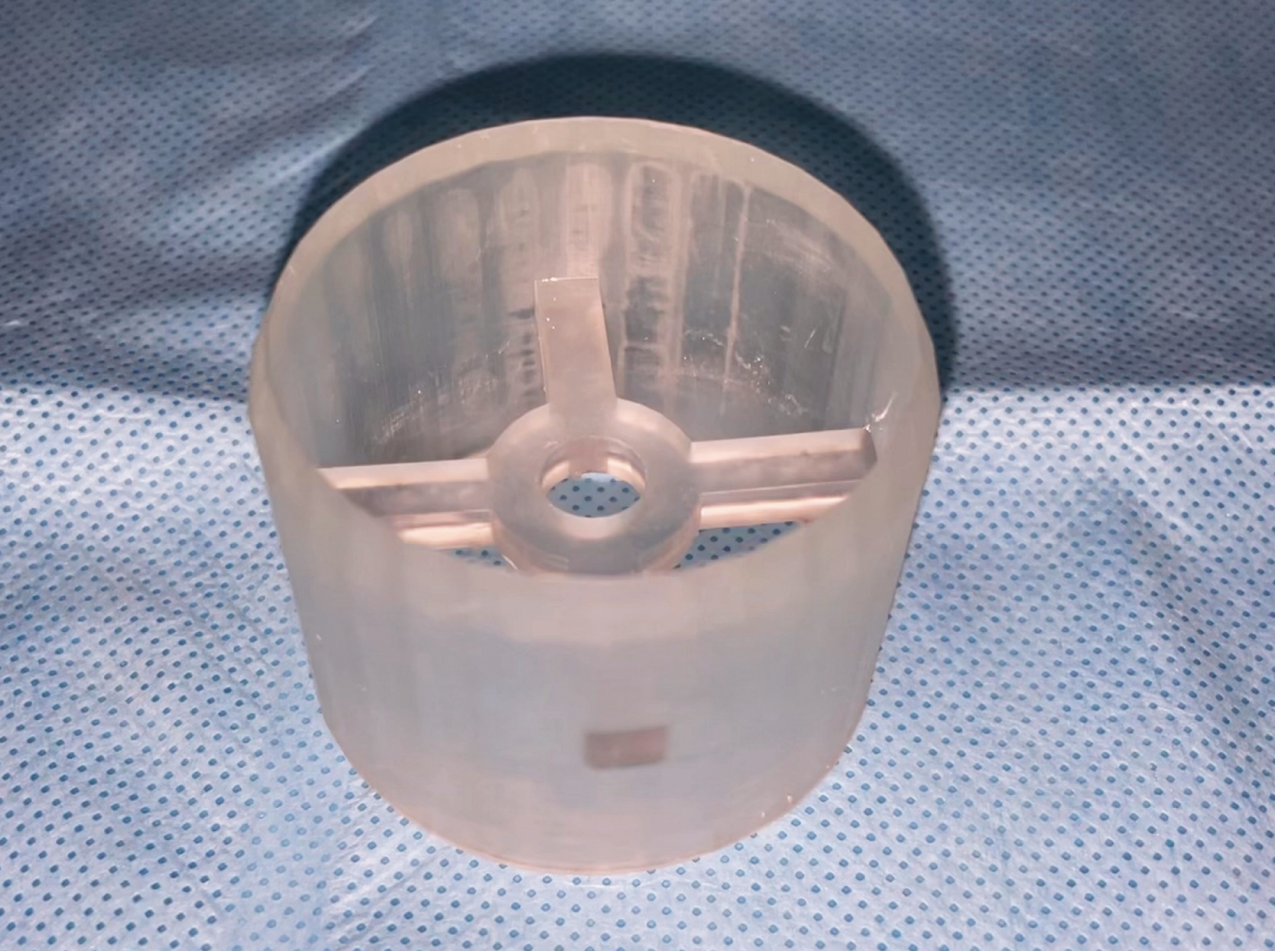
The three-dimensional printed cookie cutter model (size 42 mm).

**Figure 5.**
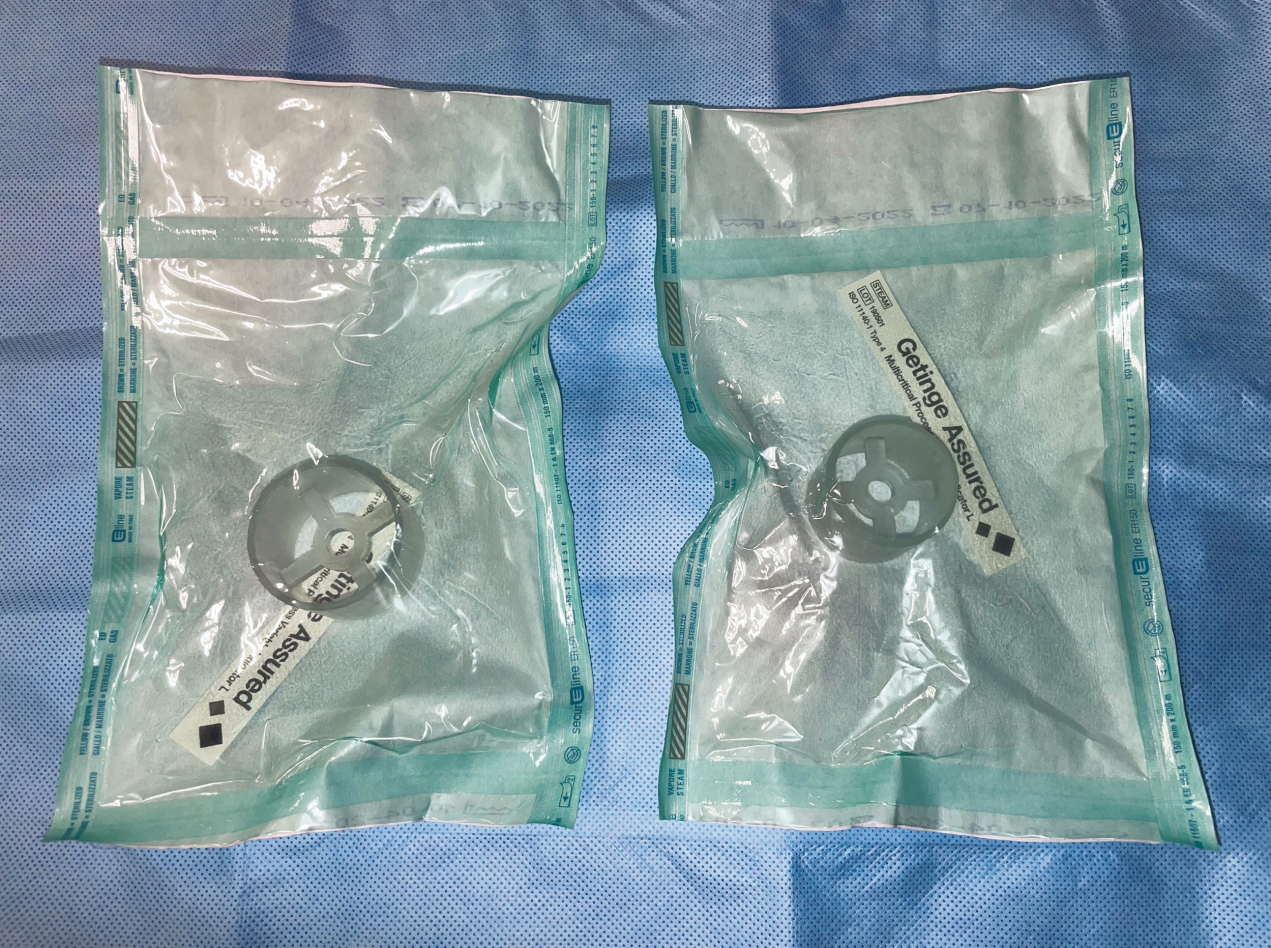
The three-dimensional printed models following sterilization with autoclave.

We created a total of 3 areola cookie cutters of 3 different sizes (33, 38, and 42 mm) (Figures 3, 4). The type of resin material used was BioMed Clear resin. The models were able to withstand sterilization in an autoclave (Figure 5).

## DISCUSSION

The use of 3D printing has proven to be a valuable tool in many areas of plastic surgery. Its widespread use in surgical planning, simulation, patient education, and the customization of prosthesis has revolutionized the field.^1^ Although a number of ethical implications should be taken into consideration, eg, patient consent and conflicts of interest, the benefits of 3D printing in delivering patient-specific care as well as improving patient satisfaction cannot be overlooked.^1^

Despite its numerous advantages, there is paucity in the literature on the use of 3D printing to create surgical instruments.^2,7-10^ Combining the qualities of a quick production time, accuracy in design, and ease of modification according to both the surgeon and patient’s preferences, the 3D printing of surgical instruments and intraoperative guides could also play a role in the provision of surgical care in low-resource areas.^12^

The field of plastic surgery provides an environment that fosters creativity and improvisation, and several methods have been described in the literature as a replacement for the classical cookie cutter.^13-17^ With the availability of a 3D printing facility at our hospital, we describe our successful experience in designing and printing an areola cookie cutter that is compatible with the process of sterilization.

Various innovative methods have been implemented in the literature to create custom areola markers, from washer sets to wires, electrocardiography dots, and wavy line markers.^13-17^ As evident by the literature, a symmetrical nipple-areola complex (NAC) plays a pivotal role in patient satisfaction.^18^ As a result, the importance of accurate and symmetrical measurements of a patient’s NAC dimensions cannot be overstated, making cookie cutters superior to the previously mentioned improvisations.

The commonly used cookie cutter sets are manufactured with a limited range of sizes (between 38 and 45 mm),^19^ this coincides with the most common diameter of female NACs (mean of 40 mm).^20^ Inevitably, this excludes a subset of patients where their desired or ideal NAC dimensions are less than what the commercially available cookie cutters provide. This includes male patients with severe gynecomastia or those undergoing female-to-male gender-affirming surgeries; in that cohort of patients, the ideal NAC dimensions are that of a male (mean areola diameter 25.9 mm).^21^ In order to mark the anticipated NAC dimensions, surgeons are here left with no choice but to improvise both preoperatively during patient counseling and intraoperatively.^22^ This creates room for imprecision and compromises accuracy.

This dilemma can be overcome by utilizing 3D printing; the relative cost-effectiveness, quick production time, and accuracy in design allow the creation of patient-specific areola markers of various sizes. These can subsequently be used in both preoperative counseling and intraoperatively.

The materials used in 3D printing vary widely; choosing a material that has been cross-tested for clinical use is paramount. In our experience, we have decided upon this type of resin material due to its biocompatibility and compliance with International Organization for Standardization (ISO) standards, as it is successfully tested in terms of cytotoxicity (ISO 10993-5), irritation and sensitization (ISO 10993-10), as well as mutagenicity (ISO 10993-3), which proves it is suitable for extended skin and mucosal contact.^23^ In addition, BioMed Clear Resin is ISO 13485 certified and is included in an American FDA Device Master File.^24^ Furthermore, the design can be extrapolated to other materials, which have been previously used for the production of surgical instruments (eg, poly-lactic acid).^7-9,25^

## CONCLUSIONS

Alongside its numerous applications, in this report, we highlight the role of 3D printing in successfully creating a surgical instrument. The quick production time and accuracy of the printing process are worthy of note. Despite the multiple advantages, unlimited capabilities, and invaluable contribution of 3D printing to the field of medicine, further studies are required to ascertain the long-term safety, precision, and cost-effectiveness of the 3D-printed surgical instruments against commercially available instruments.

## References

[1] Lynn AQ, Pflibsen LR, Smith AA, Rebecca AM, Teven CM. Three-dimensional printing in plastic surgery: current applications, future directions, and ethical implications. Plast Reconstr Surg Glob Open. 2021;9(3):e3465. doi: 10.1097/gox.0000000000003465 33968548 PMC8099403

[2] George M, Aroom KR, Hawes HG, Gill BS, Love J. 3D printed surgical instruments: the design and fabrication process. World J Surg. 2017;41(1):314-319. doi: 10.1007/s00268-016-3814-5 27822724 PMC6287965

[3] Krauel L, Valls-Esteve A, Tejo-Otero A, Fenollosa-Artés F. 3D-Printing in surgery: beyond bone structures. A review. Annals 3D Print Med. 2021;4:100039:1-7. doi: 10.1016/j.stlm.2021.100039

[4] Kholgh Eshkalak S, Rezvani Ghomi E, Dai Y, Choudhury D, Ramakrishna S. The role of three-dimensional printing in healthcare and medicine. Mater Des 2020;194:108940:1-15. doi: 10.1016/j.matdes.2020.108940

[5] Puls N, Carluccio D, Batstone MD, Novak JI. The rise of additive manufacturing for ocular and orbital prostheses: a systematic literature review. Annals 3D Print Med. 2021;4:100036:1-6. doi: 10.1016/j.stlm.2021.100036

[6] Burnard JL, Parr WCH, Choy WJ, Walsh WR, Mobbs RJ. 3D-printed spine surgery implants: a systematic review of the efficacy and clinical safety profile of patient-specific and off-the-shelf devices. Eur Spine J. 2020;29(6):1248-1260. doi: 10.1007/s00586-019-06236-2 31797140

[7] Kondor S, Grant CG, Liacouras P, et al On demand additive manufacturing of a basic surgical kit. J Med Dev. 2013;7(3):030916. doi: 10.1115/1.4024490

[8] Chen JV, Dang ABC, Lee CS, Dang ABC. 3D printed PLA Army-Navy retractors when used as linear retractors yield clinically acceptable tolerances. 3D Print Med. 2019;5(1):16. doi: 10.1186/s41205-019-0053-z 31754879 PMC6873412

[9] Zaidi S, Naik P, Ahmed S. Three-dimensional printed instruments used in a Septoplasty: a new paradigm in Surgery. Laryngoscope Investig Otolaryngol. 2021;6(4):613-618. doi: 10.1002/lio2.579 PMC8356858 34401479

[10] Rankin TM, Giovinco NA, Cucher DJ, Watts G, Hurwitz B, Armstrong DG. Three-dimensional printing surgical instruments: are we there yet? J Surg Res. 2014;189(2):193-197. doi: 10.1016/j.jss.2014.02.020 24721602 PMC4460996

[11] Formlabs. Biomed Clear Resin. Updated September 2, 2020. Accessed March 18, 2022. https://formlabs-media.formlabs.com/datasheets/2001432-TDS-ENUS-0.pdf

[12] Ibrahim AMS, Jose RR, Rabie AN, Gerstle TL, Lee BT, Lin SJ. Three-dimensional printing in developing countries. Plast Reconstr Surg Globl Open. 2015;3(7):e443. doi: 10.1097/gox.0000000000000298 PMC4527617 26301132

[13] Mahajan AL, Riordan CL, Hussey AJ, Regan PJ. The electrocardiography dot as a preoperative marker for nipple-areola complex reconstruction. Plast Reconstr Surg. 2003;111(2):955. doi: 10.1097/00006534-200302000-00095 12560741

[14] Lanfranchi LA, Gazzola R, Preis FWB. Useful method to create a precise, sterile, and inexpensive areola marker. Aesthet Surg J. 2013;33(6):922-923. doi: 10.1177/1090820X13494758 23908308

[15] Cek DI . Custom-made periareolar wavy-line marker. Plast Reconstr Surg. 2004;113(1):454-455; author reply 455. doi: 10.1097/01.PRS.0000100610.69566.8F 14707683

[16] Köse AA, Karabağli Y, Çetin C. A new areola marker. Plast Reconstr Surg. 2005;115(5):1452-1453. doi: 10.1097/01.prs.0000157641.52142.5d 15809637

[17] Stocchero NI . An areola marker. Plast Reconstr Surg. 2003;111(5):1776-1777. doi: 10.1097/00006534-200304150-00049 12655247

[18] Goh SCJ, Martin NA, Pandya AN, Cutress RI. Patient satisfaction following nipple-areolar complex reconstruction and tattooing. J Plast Reconstr Aesthet Surg. 2011;64(3):360-363. doi: 10.1016/j.bjps.2010.05.010 20570584

[19] Hauben DJ, Adler N, Silfen R, Regev D. Breast–areola–nipple proportion. Ann Plast Surg. 2003;50(5):510-513. doi: 10.1097/01.SAP.0000044145.34573.F0 12792541

[20] Yue D, Cooper LRL, Kerstein R, Charman SC, Kang NV. Defining normal parameters for the male nipple-areola complex: a prospective observational study and recommendations for placement on the chest wall. Aesthet Surg J 2018;38(7):742-748. doi: 10.1093/asj/sjx245 29329370

[21] Maas M, Howell AC, Gould DJ, Ray EC. The ideal male nipple-areola complex: a critical review of the literature and discussion of surgical techniques for female-to-male gender-confirming surgery. Ann Plast Surg. 2020;84(3):334-340. doi: 10.1097/SAP.0000000000002018 31633544

[22] Aksam E, Aksam B, Demirseren ME. A practical way for nipple-areola complex reshaping in circumareolar reduction of gynecomastia. Aesthet Surg J 2015;35(6):NP186-NP187. doi: 10.1093/asj/sjv035 26069154 PMC4520583

[23] Formlabs Ohio, Inc. Certifications and standards of Formlabs resins. Published online April 11, 2022. https://support.formlabs.com/s/article/Certifications-and-standards?language=en_US

[24] US Food & Drug Administration. Establishment Registration & Device Listing. Updated July 4, 2022. Accessed April 11, 2022. https://www.accessdata.fda.gov/scripts/cdrh/cfdocs/cfRL/rl.cfm?rid=228268

[25] Alghounaim M, Almazeedi S, Al Youha S, et al Low-cost polyester-tipped three-dimensionally printed nasopharyngeal swab for the detection of severe acute respiratory syndrome-related coronavirus 2 (SARS-CoV-2). McAdam AJ, ed. J Clin Microbiol. 2020;58(11):e01668-20. doi: 10.1128/JCM.01668-20 PMC7587115 32817232

